# Innovative strategies for pollution assessment in Northern Bangladesh: Mapping pollution areas and tracing metal(loid)s sources in various soil types

**DOI:** 10.1371/journal.pone.0311270

**Published:** 2025-02-03

**Authors:** Abdullah Al Yeamin, Md. Yousuf Mia, Shahidur R. Khan, M. Safiur Rahman, Venkatramanan Senapathi, Abu Reza Md. Towfiqul Islam, Tasrina Rabia Choudhury

**Affiliations:** 1 Department of Disaster Management, Begum Rokeya University, Rangpur, Bangladesh; 2 Chemistry Division, Analytical Chemistry Laboratory, Atomic Energy Centre Dhaka, Bangladesh Atomic Energy Commission, Dhaka, Bangladesh; 3 Chemistry Division, Water Quality Research Laboratory, Atomic Energy Centre Dhaka, Bangladesh Atomic Energy Commission, Dhaka, Bangladesh; 4 PG and Research Department of Geology, National College (Autonomous), Tiruchirappalli, Tamil Nadu, India; 5 Department of Development Studies, Daffodil International University, Dhaka, Bangladesh; 6 Department of Earth and Environmental Science, College of Science, Korea University, Seongbuk-gu, Seoul, Republic of Korea; Ardakan University, ISLAMIC REPUBLIC OF IRAN

## Abstract

This study assessed the risks of soil pollution by heavy metals in Chilmari Upazila, northern Bangladesh, using the static environmental resilience (Pi) model of soil. Geostatistical modeling and self-organizing maps (SOM) identified pollution areas and spatial patterns, while a positive matrix factorization (PMF) model revealed pollution sources. The results showed that the average concentrations of Cr, Pb and As were well above background levels. Agricultural and industrial soils were mainly contaminated with Cr, Pb and As according to the Nemerow Pollution Index (NPI), Ecological Risk (ER) and Pi Index. Over 70% of the sites were contaminated with Pb and Cr, while co-contamination was particularly high. A one-way ANOVA showed significant correlations between Pb, Cu and Zn levels and human activities. The PMF analysis revealed that industrial effluents, agrochemicals and lithogenic sources were the main contributors to soil contamination with 16%, 41% and 43%, respectively. The SOM analysis revealed three distinct spatial patterns (Pb-Zn, Cr-Cu-Ni and Co-Mn-As), which are consistent with the PMF results. These results emphasize the need for stringent measures to reduce industrial emissions and remediate soil contamination in order to improve soil quality and food security.

## 1. Introduction

The process of industrialization and urbanization is leading to soil degradation worldwide, with developing countries such as Bangladesh experiencing significant impacts [1,2]. Research by Hasan et al [3] shows that about 20% of soils in Bangladesh, including 30% of agricultural soils, are contaminated with heavy metals. Due to their high ecotoxicity, long-term persistence and accumulation in the food web, soil contamination with heavy metals is problematic. This threatens agricultural ecosystems and food safety. Industrial activities, vehicle emissions and agrochemicals release significant amounts of metal(loid)s into the soil environment [4]. This leads to contamination of the soil with metals [5]. High concentrations of heavy metals in soil threaten human and ecological health [6]. Heavy metals in contaminated soils can quickly enter the human body through the ingestion of plants consumed as food and thus pose a direct health risk. In addition, these pollutants can damage soil microorganisms, reduce the nutritional content of plants and, depending on the form and amount of the substance, potentially lead to health problems such as cancer, behavioral disorders and organ dysfunction [7,8]. Excessive levels of Pb can damage the central nervous system when ingested, causing headaches, insomnia and memory loss [9]. High Cr intake can lead to gastrointestinal problems or death [10,11]. Plant roots can absorb Cu from the soil and harm human health [12]. Therefore, an in-depth study of the risks of heavy metals to humans from various land uses has become an important topic of modern environmental science, which is of paramount importance for the protection of human health and the promotion of sustainable development.

To protect soil ecology and human health, the simple pollution index, Nemerow’s integrated pollution index, and the geoaccumulation index have been proposed to analyze metal pollution in soil [13,14]. However, these indices only analyze the basic pollutant levels of the environmental quality standards offered for soils. However, the Environmental Pollution Capacity (EPC) is the highest amount of toxic material that a system can absorb without polluting the environment [15]. It is used extensively in hydro-atmospheric systems to set local limits on total pollutant emissions [16]. The environmental quality standard for soils defines the EPC of soils as the maximum number of pollutants in the soil without compromising agricultural productivity or quality. Previous research on the ecological capacity of soils has defined threshold values and safety equivalents, developed assessment approaches, created analytical frameworks and developed prediction methods [17]. The ecological capacity of soil for heavy metal (loids) still needs to be thoroughly analyzed for different land use patterns. The ecological capacity of the soil for heavy metals can therefore be an indicator of metal pollution and provide clues for the prevention and reduction of metal inputs.

The spatial heterogeneity of metal (loid) concentrations in the soil caused by human activities makes it difficult to identify regional contamination sites [5,18]. Time and money hinder the control and remediation of all areas affected by heavy metal(loid)s. A systematic and sequential management plan for the most important pollution sites is more feasible if it relates to the area of heavy metal pollution. GIS is generally used to present spatial visualization and diversity in pollution assessment [19] and to identify contaminated sites. Most studies have used a geostatistical interpolation approach to identify metal-polluted areas [20,21]. However, pollution assessment and interpolation processes are subject to uncertainties, such as the source and characteristics of the data and kriging interpolation [22]. Xia et al. (2024) [23] investigated metal concentrations and local geographic variability to determine interpolation errors. Smoothing effects can change local high/low values during interpolation and thus affect the identification of contaminated areas. Therefore, geostatistical modeling is required to analyze and reduce uncertainty in the identification of contaminated areas in order to make decisions about soil remediation.

Index methods such as the Nemerow Pollution Index (NPI), the Contamination Factor (CF), the Pollution Load Index (PLI) and the Potential Ecological Risk Index (ERI) have been used in numerous studies to estimate the risk of heavy metal contamination in soil. These methods have proven to be helpful in managing and mitigating soil pollution risks [1,24]. The mobilization and alteration of heavy metals in soil depends on physico-chemical factors that can be influenced by changes in land use. Different land use patterns lead to different levels and sources of metal pollution, which affect the soil environment and cause pollution problems [25]. Previous research has focused on pollution hazards from specific land use categories in agricultural, mining and urban areas [26–28]. However, detailed studies on soil pollution in river ports are lacking. As different land use patterns lead to different soil pollution, it is crucial to prevent and control heavy metal pollution in heavily populated regions, to identify the sources of heavy metals in soil and to determine their contribution.

Identifying and attributing the source of heavy metal(loid)s is crucial for the control and elimination of soil contamination. Several quantitative methods have been used to determine the possible sources of heavy metal(loid)s in soil [29]. For example, chemometric methods such as principal component analysis (PCA), factor analysis and correlation analysis have been widely applied to identify the sources of heavy metals [9,30]. Due to the drawbacks in dealing with the type of contaminated soil, sources and contribution fraction, the US Environmental Protection Agency (EPA) has adopted the positive matrix factorization (PMF) method. The advantages of PCA are the non-negativity constraint, number of sources, and source uncertainty [20]. The PMF method has been used to determine the sources of heavy metal pollution in agricultural, urban and floodplain soils [23,28,31]. PMF results are rarely used to assess geographic characteristics for source variables, allowing the investigation of factors influencing metal pollution patterns. In addition to the PMF model, self-organizing maps (SOM), an unsupervised machine learning model for explanation and visualization, are currently used to visualize high-dimensional data in low-dimensional space [32]. SOM can extract rich information from complicated datasets to categorize and recognize patterns, and PMF can verify the results of SOM in the distribution of heavy metal (loid)s [33]. These approaches have been used separately to analyze source apportionment, but their combined use has yet to be documented [34]. The combination of geostatistical, SOM and PMF models could provide sufficient information to guide source management and remediation measures.

The socio-economic strength of northern Bangladesh has increased in recent decades due to rapid urbanization and traffic-free zones in the Chilmari Upazila (sub-district—the second largest administrative division), which has a long tradition as a river port and agricultural center [35]. With accelerated economic and social development, brick factories, dyeing factories and small businesses have released pollution. This has led to an accumulation of heavy metal (loid) metals in the soil, which can harm ecosystems and public health. As the economy is growing, metal pollution in the soil threatens food security in this upazila [36]. Rahman et al (2015) found that local emissions of agrochemicals lead to severe soil degradation and heavy metal pollution in the study area. Human-induced soil contamination complicates the assessment of pollution risks and the identification of contaminated areas in different land use patterns [37].

This study assesses pollution risk, identifies zones of heavy metal contamination and identifies potential sources of pollution in a rapidly growing region in order to propose sustainable soil management strategies. Thorough research and comprehensive analysis will improve soil quality and support community development. The main objectives are: (i) to assess the risk of pollution from heavy metals using a soil environmental capacity model; (ii) to delineate pollution hotspots and address uncertainties through geostatistical modeling; (iii) to investigate the spatial distribution patterns of heavy metal content using a SOM; and (iv) to map potential pollution sources and their spatial characteristics using the PMF model. The results will improve pollution assessment, more accurately recognize pollution areas and identify sources, contributing to effective pollution management and remediation strategies for contaminated soils.

## 2. Materials and methods

### 2.1. Location of the study area

**Fig 1:**
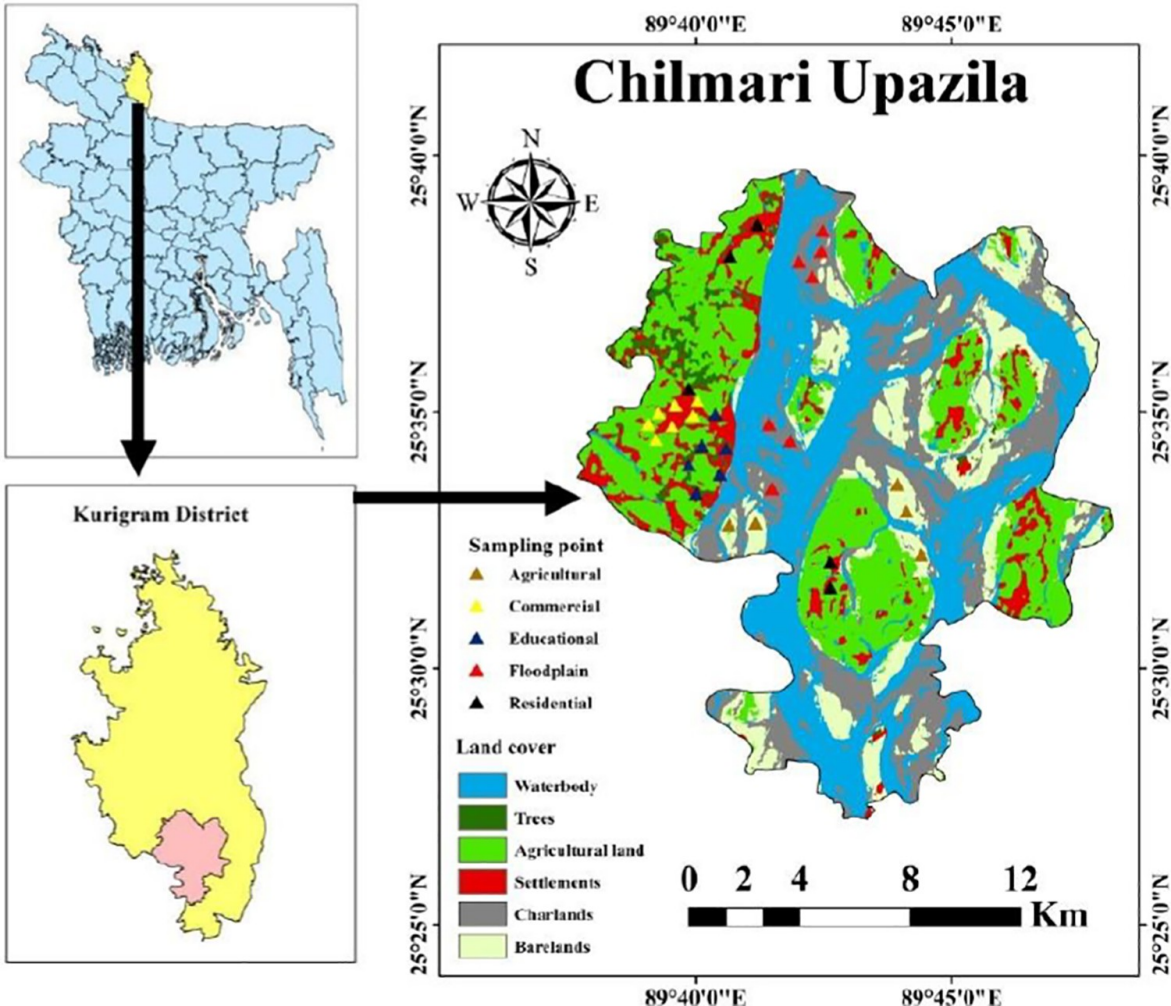
Location of the study area with sampling points.

The area selected for this study is in Chilmari Upazila, a major river port in northern Bangladesh near the border with India. This sub-district lies on the western bank of the Jamuna River, known as the Brahmaputra as it enters Bangladesh from India, and is of strategic importance to the region (Fig 1). The government has announced the reopening of the Chilmari river port in response to a long-standing demand from local residents. The area under study has a major impact on economic development in northern Bangladesh due to the extensive use of chemical fertilizers and the region’s heavy dependence on the Brahmaputra River. Topographically and geomorphologically, the area is located in the Teesta floodplain in northern Bangladesh, which is typical of the Brahmaputra basin. Overall, the soil layer in the region is generally thin and loamy. In the northern and central parts of the areas, floodplains were the predominant type of land use.

The study area is characterized by a subtropical monsoon climate. The average annual temperature is 25°C and the rainfall is 2500 mm. Loose alluvial sediments are gradually deposited by the river bed and its tributaries make up the bulk of the soil composition. Due to the seasonal fluctuations of the river and the nature of the floodplain, the region is known for regular flooding. Although there is no official industrial area, Chilmari Upazila makes a significant economic contribution to the country. The major industries are brickworks, coking plants, farms, small industries etc. These industrial activities are closely related to the pollution of soil in Chilmari Upazila by heavy metals.

### 2.2. Sample collection and processing

In December 2023, a total of 30 soil samples were collected from the Chilmari Upazila in northern Bangladesh, covering the five typical land use types, i.e. residential areas (n = 5), commercial areas (n = 7), educational areas (n = 6), floodplains (n = 7) and agricultural areas (n = 5). The area is divided into strata based on land use types and samples are taken from each stratum. Two subsamples were mixed and taken at least one meter from each sampling point to create a single composite soil sample (0–10 cm depth). After soil samples were then dried in a cool, well-ventilated room and packed in a polyethylene bag before transportation to the laboratory.

The soils were then placed in Petri dishes and placed in the oven at 105°C to dry. The purified samples were homogenized using an agate mortar. Before further chemical analysis and digestion on the hot plate, all tissues were stored in a zippered polyethylene bag. The study was conducted at Analytical Chemistry Laboratory (ISO/IEC 17025:2017 accredited, Department of Chemistry, Atomic Energy Center Dhaka, Bangladesh Atomic Energy Commission). This laboratory has developed techniques for the analysis of heavy metals in soil samples, including soil digestion, and these techniques have been accredited by the competent authority and comply with the ISO/IEC 17025 standard. This method ensures the accurate and reliable determination of heavy metals in soil samples.

### 2.3. Analytical processes and quality check

The soil samples were first air-dried and then ground so finely that they passed through a 0.149 mm nylon sieve. An accredited laboratory method was then used to digest the soil samples using a hot plate. Dry soil samples weighing 0.5 g were placed in 100 ml beakers and treated with 10 ml aqua regia. After heating on a hot plate for at least 8 hours to eliminate oxidizable substances, the digested solutions were filtered with filter paper and DI water and then stored in 25 ml vials. Throughout the digestion process, comparative analyzes were performed with blanks, duplicates and spike samples. The criteria for the selection of metals such as Pb, Cr, Mn, Cu, Zn, Co, Ni and As in soil samples are primarily based on their environmental and health impacts, regulatory standards, sources of contamination, historical data, toxicity, mobility and potential ecosystem impacts. This comprehensive approach ensures that soil quality is effectively monitored and managed to protect human health and the environment. It is noted that Cd was analyzed, but all values are below the detection limit (< 0.002 mg/kg). Therefore, Cd was not considered for this study.

After digestion, the concentrations of heavy metals (Cr, Ni, Cu, Zn, Mn, Pb and Co) were determined using a Varian AA240FS atomic absorption spectrometer in flame mode. Arsenic was detected using a Varian AA280Z atomic absorption spectrometer (AAS) with Zeeman background correction system, equipped with a graphite furnace (GTA 120) and an autosampler (PSD 120). Working standard solutions were prepared by diluting the corresponding 1000 mg/L stock standard solutions (SpectroPure, USA) with 1% (w/w) high purity nitric acid (Merck, Darmstadt, Germany).

Quality assurance and quality control (QA/QC) measures included the use of certified reference material (soil-based matrices) and duplicate analyzes of 10% of the samples. Analysis showed excellent recoveries for blank, replicate and spike samples, with recoveries between 90 and 110% with a relative standard deviation (RSD) within 10%. Each analysis was performed in triplicate for both the samples and the diluted standard solutions, aiming for an average RSD of less than 10%, which was considered acceptable. Quality control (QC) samples were regularly included to ensure analytical consistency, and the limit of detection (LOD) was established during method validation [25]. The certified standard reference materials for soil analysis are IAEA-33 and IAEA-SL-1, which were used for these analyzes. In addition, a control chart was maintained for each analysis to monitor deviations from the QC baseline and ensure analytical integrity. The detection limits were set at 0.25, 0.21, 0.16, 0.15, 0.54, 0.12 and 0.05 mg/kg for Pb, Zn, Cr, Mn, Ni, Co and Cu and 0.3μg/kg for As.

### 2.4. Methods

#### 2.4.1. Environmental capacity assessment model

The range normalization method was used to calculate the residual ecological capacity of the different elements in the study area [23,38]. To emphasize the importance of low EPC, an improved comprehensive Nemerow pollution index and an ecological risk index were used. The calculation formula to illustrate the EPC is as follows: Eqs (1–3):

$$P_{i}=\frac{Q_{i}}{Q_{ih}}$$ (1)

$$Q_{i}=10^{-6}\times M\times (C_{b}-C_{n})$$ (2)

$$Q_{ib}=10^{-6}\times M\times (C_{b}-C_{n})$$ (3)

Pi stands for the EPC index for metal. *Q*_*i*_ and *Q*_*ib*_ stand for the existing and total EPC. M stands for the soil weight per hectare of arable land; Cs stands for the risk screening value for metal; *C*_*n*_ and *C*_*n*_ are identified concentrations and background values for metal(loid)s. S1 Table in S1 File shows the thorough assessment and classification of heavy metals in the different land uses in the study area.

The Nemerow pollution index takes into account high values or exceptional values that are above average. It is a balanced multifactorial environmental quality indicator [39]. The following Eq 4 is used to calculate the comprehensive pollution index.

$$NPI=\frac{C_{n}}{S_{j}}$$ (4)

where NPI is the Nemerow Pollution Index for a single metal at a single site and *S*_*j*_ is the standard limit value for metal in soil and *C*_*n*_ is the concentration for metal in soil.

The potential ecological risk index (ER) was formulated to assess the extent of heavy metal (loid) pollution in soil, taking into account their toxicity and environmental capacity. The ecological risk assessment of heavy metal(loid)s was performed according to the method proposed by Hakanson [40] Eqs 5 and 6.

$$ER=T_{r}\times CF$$ (5)

$$CF=\frac{C_{n}}{C_{b}}$$ (6)

ER is the ecological risk factor for a single element, *T*_*r*_ is the toxic response factor for a specific substance; CF stands for the contamination factor of heavy metals. The risk screening values, the global geochemical background values, the values for the toxic response factor and the default limits for heavy metals are listed in S2 Table in S1 File.

#### 2.4.2. Self-organizing map (SOM)

Self-Organizing Maps (SOM) are a data visualization method that aims to condense dimensions into subspaces. Within these subspaces, the similarity between data points is determined by their geometric associations [19,32]. As an unsupervised artificial neural network (ANN), SOM can make predictions, classify and create clusters in different domains [33]. By training an ANN to mirror the patterns of the input data while preserving their topo-metric properties, SOM clusters similar patterns while removing dissimilar patterns [41]. Research has shown that SOM can mitigate the shortcomings of conventional classification methods [42]. The application of the SOM method was performed using MATLAB.

#### 2.4.3. Positive matrix factorization (PMF) method

The PMF method, originally conceived by Paatero & Tapper (1994) [43] and subsequently refined by the US EPA, is considered a mathematical framework for analyzing a wide range of sample data, ranging from sediment composition to atmospheric elements [44]. Extensive studies have reliably confirmed the effectiveness of the PMF in identifying the sources of heavy metals and quantifying their respective contributions [26]. In addition, the PMF has proven invaluable in formulating targeted pollution control measures. It fulfills a dual function: it shortens complicated data sets to different source types and assesses the associated uncertainties [20]. When applied to soil environments, this methodology treats the data set as a matrix (X) containing estimated samples (n) and estimated natural species (m). The model aims to estimate the number of factors (p), the species profile for each factor (f) and the mass contribution (g) of each factor to each sample, as described in Eq 7 [45].

$$X_{ij}=\sum_{k=1}^{p}g_{ik}f_{kj}+e_{ij}$$ (7)

Where *X*_*ij*_ is the content of metal(loid)s j at the ith sampling point; p is the pollution factor; *g*_*ik*_ is the factor contribution matrix; *f*_*kj*_ is the factor element matrix and *i*_*ij*_ is the residual error matrix.

The objective function Q is employed to compute the following Eq 8, aimed at determining the optimal solution for the number of source factors utilizing the weighted least squares method:

$$Q=\sum_{i=1}^{n}\sum_{j=1}^{m}{\left(\frac{e_{ij}}{u_{ij}}\right)}^{2}$$ (8)

where n is the number of sampling points, m is the number of heavy metal(loid)s and uij is the uncertainty calculated according to Eq 9:

$$\left\{ \begin{array}{ll} \frac{5}{6}\times MDL & \mathrm{if}\;x_{ij}\leq MDL\\ \sqrt{{\left(\sigma_{j}\times x_{ij}\right)}^{2}+(0.5\times MDL{)}^{2}} & \mathrm{if}\;x_{ij}>MDL \end{array}\right.$$ (9)

where *σ*_*j*_ is the comparative standard deviation (STD) of metal(loid)s, *x*_*ij*_ is the metal(loid)s concentration and MDL is the detection limit for metal(loid)s. The MDL values are shown in Table S3. PMF version 5.0 (US EPA, 2014) was used in this study.

#### 2.4.4. Statistical and spatial analysis

The statistical analyzes, e.g. descriptive statistics, correlation and ANOVA tests, were performed with IBM SPSS 25 (SPSS Inc., Chicago, IL, USA). Prior to the one-way ANOVA and geostatistical analyzes, a normality test (Kolmogorov-Smirnov test) was performed to assess the normality of the original soil datasets. The Box-Cox transformation was used to normalize the original soil datasets that did not conform to the normal distribution. Differences in heavy metal (loid) held were compared between land use types using ANOVA. Empirical Bayesian Kriging (EBK) was chosen as the spatial analysis technique for this study. In contrast to classical kriging, EBK takes into account the error associated with the estimation of the semi-variogram model. The procedural steps were as follows: First, a semivariogram model was calculated based on the soil heavy metal contamination data. Then, a new value was simulated for each input data item using the semivariogram. A new semivariogram model was then derived from the simulated data, followed by calculating the weight of this semivariogram using Bayes’ rule to show the potential of generating observations from a semivariogram [46]. ArcGIS 10.8 was used to perform the FBC interpolation and visualize the regional distributions of heavy metals in soil.

## 3. Results and discussion

### 3.1 Characteristics of heavy metal(loid) levels in soil and assessment of contamination under different land use types

The basic statistical analysis (e.g. maximum, minimum, mean, standard deviation) of eight heavy metal(loid)s (Pb, Cr, Mn, Cu, Zn, Co, Ni and As) in five land use patterns is shown in Table 1.

**Table 1:** Descriptive statistics of heavy metal(loid)s in different land use patterns.

	Pb	Cr	Mn	Cu	Zn	Co	Ni	As
**Residential Area**								
Max.	172.65	194.17	367.06	21.86	146.28	17.69	29.00	32.18
Min.	64.10	13.02	269.61	10.82	58.51	8.40	16.90	16.65
Mean	115.28	67.74	320.69	17.09	98.54	13.97	22.56	25.23
STD.	38.96	72.04	42.16	4.17	36.10	3.58	4.42	6.00
**Commercial Area**								
Max.	158.28	95.02	362.31	30.97	160.61	20.22	34.13	69.43
Min.	63.37	43.37	263.95	14.61	31.32	13.90	20.14	19.58
Mean	105.33	72.19	316.79	22.51	61.22	17.05	26.51	32.82
STD.	28.30	16.60	42.51	6.68	45.70	2.06	5.34	16.77
**Educational Area**								
Max.	239.77	236.48	366.37	24.67	236.50	18.71	28.47	43.64
Min.	124.12	77.80	223.79	13.50	49.35	5.29	19.54	18.39
Mean	155.77	141.68	300.58	19.97	92.80	11.30	24.35	27.89
STD.	42.19	63.21	48.46	4.40	71.61	5.11	3.78	9.37
**Floodplain Area**								
Max.	140.14	2416.24	746.36	80.62	20.72	7.02	51.83	24.69
Min.	95.45	166.20	155.02	8.04	10.82	3.70	14.99	11.95
Mean	120.96	553.68	280.77	21.54	15.21	5.17	22.49	16.88
STD.	16.52	825.53	207.95	26.17	3.44	1.20	13.01	4.68
**Agricultural Area**								
Max.	205.86	2416.24	746.36	80.62	236.50	20.22	51.83	69.43
Min.	151.71	13.02	42.16	4.17	3.44	1.20	3.78	4.68
Mean	173.71	280.02	286.70	21.50	64.68	10.92	23.57	28.33
STD.	23.19	492.62	129.95	14.93	54.52	5.62	9.36	16.94
FAO Standard background value*	50	100	2000	100	300	50	100	20

Max.: Maximum; Min.: Minimum; STD.: Standard deviation

*Chiroma et al., (2014)[47].

The average Pb concentration in all types of land use exceeded the background value of 50 mg/kg [47,48]. Compared to the FAO background values, Pb, Cr and As showed a significant enrichment in the soils of the different land use types. The mean concentrations of Cr and Pb in agricultural soils were > 3 and 3.5 times higher than the local baseline, respectively. Zn and Cu were comparable to the background value, and the mean concentrations of Mn, Co and Ni were lower than the background value. In addition, the average concentration of Cr in the alluvial soil was significantly 5 times higher than the background value. The average As concentration was higher than the background value in all soil uses, except in alluvial soils (16.88 mg/kg). Pb exceeded the background value in the samples analyzed, followed by Cr, which showed an exceedance of 99%. About 88% of the samples exceeded the background value for As. In contrast, Cu and Zn did not exceed the background value, indicating that these two metals have little/no contamination regardless of land use. Exceedances of metal contents in soils of agricultural, floodplain and industrial areas generally indicate a significant influence of human activities [6,23].

**Fig 2:**
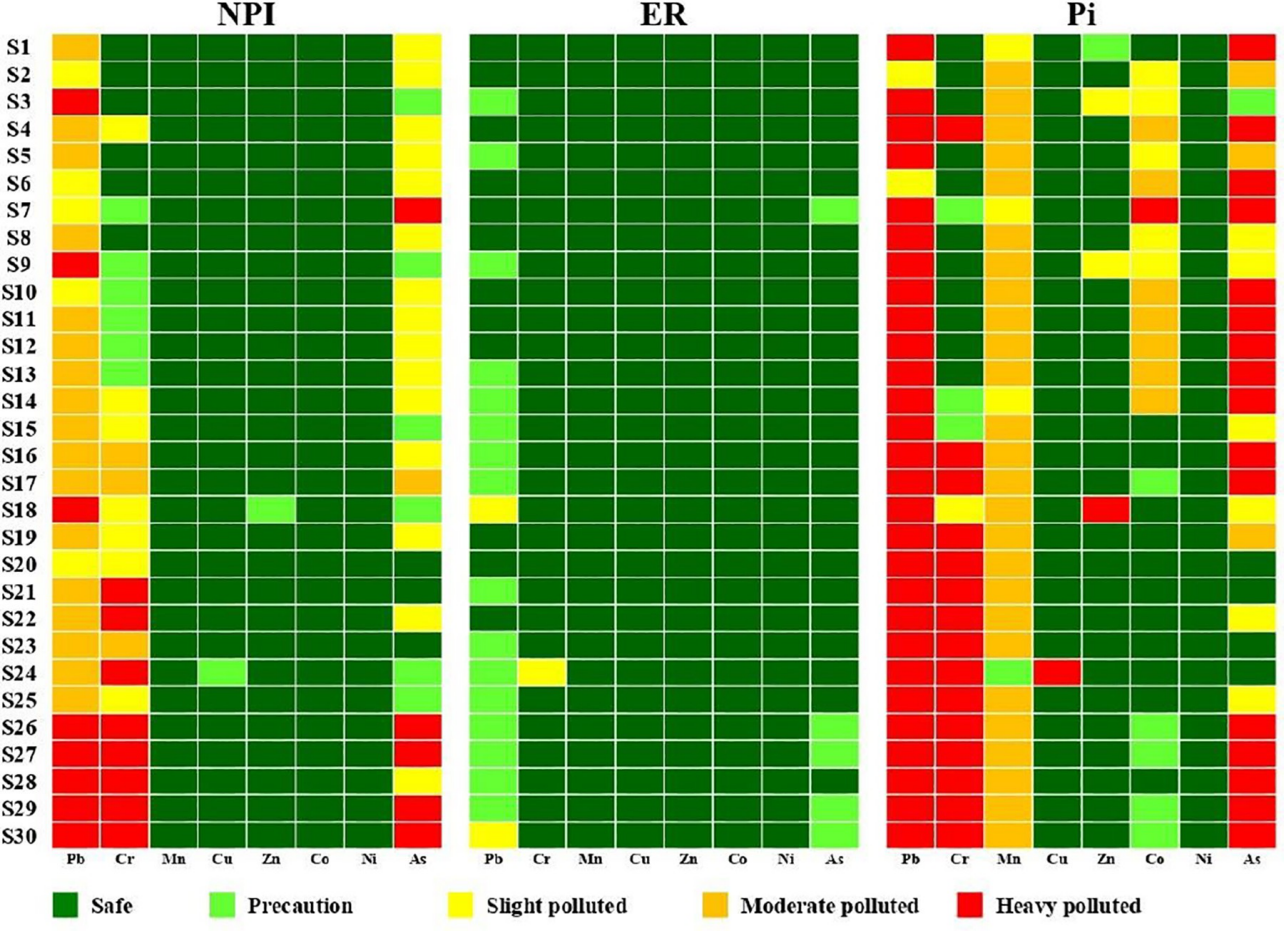
Heat maps of pollution risk based on the Nemerow Pollution Index (NPI), ecological risk (ER) and ecological carrying capacity (Pi).

The Nemerow Pollution Index (NPI), Ecological Risk (ER) and Ecological Carrying Capacity (Pi) were combined to better assess the pollution status of heavy metal(loid)s. The NPI results showed that agricultural soils are most at risk and that Pb, Cr and As are the most contaminated heavy metals (Fig 2). Only a few sites (S18 and S24) showed an increased risk of Cu and Zn contamination. At the same time, the individual contamination index showed that no site was contaminated with Ni and Cu. On the other hand, most of the sites showed a low risk for Mn and Co. Most sites had an extreme risk for Cr and Pb, and only a few sites had a medium risk for Cr.

Below you will find the average values of the ecological carrying capacity: Ni > Cu > Zn > Co > Mn > As > Pb > Cr. For Ni, Cu and Zn, an average value greater than one means that there is no risk of pollution from the heavy metals. On the other hand, values below zero for both Cr and Pb indicate an extreme risk of pollution. Negative values were also found for As at several locations, indicating an extreme risk of pollution. Furthermore, the risk of pollution from Mn and Co can be taken with a grain of salt, as the average Pi-Mn and Pi-Co values were 0.23 and 0.84 respectively.

Further investigations were carried out to evaluate the integrated EPC for all selected metal(loid)s (S4 Table in S1 File and S1 Fig in S1 File). The mean Pi values of the study area indicate a low risk of pollution. As can be seen from the distribution of Pi values, most of the sites are in the safe range (S1 Fig in S1 File). In contrast to the NPI and Pi index, the ER index identifies a larger number of sites with a low to medium risk of pollution. According to Pi, 56.67% of sites have a safe pollution risk and 20% have precautionary values. This indicates that 76.67% of the sites have a high to medium pollution capacity.

The results of heavy metal pollution analysis showed that Cr, Pb and As are the major metallic pollutants in the Chilmari Upazila as shown by Nemerow and ecological risk and ecological resilience indices. Identical results were found by Habib et al. (2023) [14] in agricultural soils in northwestern Bangladesh that were polluted by coal mining. However, the levels of Pb and Cr in the studied area were much higher than in neighboring northwestern Bangladesh [1], where small factories with heavy metal emissions have been widespread in recent decades. In addition, Siddique et al. (2020) [49] indicated low Pb contamination of coal ash samples [49]. Islam et al. (2022) [36] used the EF and ERI to assess the level of soil contamination near a large industrial area and found high accumulation of Cr and Cu in the nearby soils. Previous studies indicate that anthropogenic industrial input often triggers severe heavy metal contamination in soil [36].

Numerous cited papers have reported on the environmental impact of heavy metals in soil in various urban areas worldwide [15]. For example, Xia et al. (2024) [23] found a low EPC for metal pollution in the agricultural soils of Wenzhou in southeast China [23]. They reported that the EPC results revealed 28 sites with high to medium EPC for local heavy metal (loid) pollution. Pan et al (2021) demonstrated a low EPC for metal pollution in the northern city of Zhongshan, which was due to metal emissions from concentrated electrical processing, toy industry and hardware industry [50]. Our results were also related to those of the two areas that had low integrated capacity with low pollution risk. A maximum percentage of sites had low to excessive EPC, suggesting that metal emissions need to be addressed and the soil environment of the port area needs to be remediated [37]. Sustainable strategies should be implemented to monitor and remediate Cr, Pb and As in the port area soils, especially in agricultural and commercial soils.

### 3.2. Influences of different land use patterns on the heavy metal(loid) contents in soil

A one-way analysis of variance (ANOVA) was conducted in the study to determine whether land use patterns had an effect on heavy metals in Chilmari soils. Tukey tests were used for heavy metals (Pb, Cr, Mn, Zn and Ni) with homogeneous variance and heavy metal(loid)s (Ni, Co and As) with heterogeneous variance (Table 2).

**Table 2:** Result of the ANOVA test for soil samples between different land use patterns.

		Pb	Cr	Mn	Cu	Zn	Co	Ni	As
Residential Area	Mean	115.28	67.74	320.69	17.09	98.54	13.97	22.56	25.23
	STD	38.96	72.04	42.16	4.17	36.10	3.58	4.42	6.00
	CV%	33.80	106.36	13.15	24.41	36.63	25.60	19.59	23.79
Commercial Area	Mean	105.33	72.19	316.79	22.51	61.22	17.05	26.51	32.82
	STD	28.30	16.60	42.51	6.68	45.70	2.06	5.34	16.77
	CV%	26.87	22.99	13.42	29.69	74.64	12.08	20.12	51.09
Educational Area	Mean	155.77	141.68	300.58	19.97	92.80	11.30	24.35	27.89
	STD	42.19	63.21	48.46	4.40	71.61	5.11	3.78	9.37
	CV%	27.09	44.61	16.12	22.05	77.16	45.19	15.52	33.62
Floodplain Area	Mean	120.96	553.68	280.77	21.54	15.21	5.17	22.49	16.88
	STD	16.52	825.53	207.95	26.17	3.44	1.20	13.01	4.68
	CV%	13.66	149.10	74.06	121.51	22.60	23.28	57.86	27.76
Agricultural Area	Mean	173.71	451.25	256.77	29.59	50.40	10.99	28.95	57.10
	STD	23.19	109.80	63.65	6.66	12.43	1.95	3.91	15.93
	CV%	13.35	24.33	24.79	22.51	24.67	17.72	13.49	27.90
F		2.006	1.199	1.013	1.239	5.898	4.619	5.401	6.184
p-value		0.031	0.060	0.001	0.026	0.045	0.031	0.099	0.173

The levels of Cu, Cr and Ni were significantly higher in the soils of industrial areas, floodplains and agricultural areas than in the soils of residential areas and educational institutions, suggesting that this metal pollution was caused by agrochemical practices. In addition, the levels of Pb and Zn were higher in the soils of residential, commercial and educational areas than in the soils of agricultural and floodplain areas, suggesting that these metals originated from the sedimentation of industrial wastewater. Table 2 shows that land use had no effect on the concentrations of Cr, As and Ni. Combined with the spatial variation characteristics of these heavy metal (loid) contents, it can be concluded that the parent rock materials primarily control Co, As and Mn in the soils. However, the sources of As and Ni need to be further determined.

The Pearson correlation coefficients of eight heavy metal(loid)s are listed in S5 Table in S1 File. A significant positive correlation was observed between Pb and Zn, Pb and Cu, Pb and Mn, Mn and Cu, and Cr and As (P < 0.05) in the soils of the residential area; at the same time, Pb and Zn, Cu and Mn also showed a significant positive correlation, while Pb and Co, Zn and Co showed a significant negative correlation in the soils of the commercial area (P < 0.05). In the school soils, however, Pb and Zn, Cu and Cr as well as Cu and Ni showed a positive correlation (P < 0.01). A positive correlation coefficient between the metals in the soil indicates that these metals probably have the same source of contamination. In the present study, for example, Pb was probably highly correlated with Zn and Cr in soils from residential areas. Cu also showed a high correlation with Pb, Cr and Mn in the floodplain soils of the study, which is consistent with the results of [26]. In the agricultural soils in the study, Cu also showed a strong correlation with other heavy metals (Loid), as found in previous studies. It can be concluded that soil properties (e.g. pH, organic matter, clay, silt, sand, etc.) and the type of land use can influence the correlation of soil metals (loid) to a certain extent. It should also be noted that correlation can be beneficial in determining the positive source of land use patterns [48].

### 3.3. Spatial distribution and pollution area detection

S2 Fig in S1 File shows a map of the spatial distribution of heavy metal (loid) concentrations in soil generated by empirical Bayesian kriging interpolation (EBK). Regions with elevated Pb concentrations were predominantly located in agricultural areas and were characterized by particularly high values. Conversely, areas with elevated Cr concentrations were predominantly located in floodplains that extended in a southwesterly direction and were characterized by extensive coverage and elevated values. The distribution pattern of Mn concentrations mirrored that of Zn, both originating predominantly from industrial soil areas. Elevated As concentrations were mainly observed in regions located around agricultural areas and floodplains. Areas with high Cu and Ni concentrations are similar to those associated with As contamination. It is assumed that the soils in the agricultural areas have high Cu, Ni and As content. The Co concentration indicates that the source of Co contamination is mainly due to anthropogenic activities, including commercial and industrial soils. The concentrations of metal(loid)s are mainly due to two sources: anthropogenic and natural. Pb, Mn, Zn, Co and Ni indicate an anthropogenic source, while Cu and Cr indicate a geogenic distribution. The As includes both sources (anthropogenic and natural). The analytical results showed that the predicted distributions were good enough to describe the correct orientation of the investigated heavy metals.

With agriculture and industrial plants as the epicenter, heavy metal pollution of the soil is spreading in all directions. It shows a decreasing trend, which is consistent with the Chinese work [2]. Since kriging models are tailored to data with locally varying mean values [51], the interpolation results in this study have less non-uniform boundaries compared to conventional kriging methods, allowing a smoother transition between different concentration values [52]. In addition, parameter fitting of prediction results can mitigate non-stationary data trends, which is particularly beneficial for medium-size data sets and eliminates the need for extensive interactive modeling. Once the prior and posterior distributions of the FBC model were fixed, it showed superior performance over alternative predictors, especially in scenarios where several widely accepted assumptions on geostatistical data modeling were not met.

According to previous studies, there is a strong correlation between the duration of urbanization and the accumulation of Pb, suggesting that most Pb pollution is found in former industrial areas [12]. While the concentration distributions of Ni and Co were comparable, the overall contamination level of Co was low and that of Ni was high. Since Ni can be taken up by plants and accumulates in the human body through the food chain, special attention should be paid to Ni pollution [53]. Agricultural pollution may also contain As, Cr and Cu [44], indicating the high value of concentrations and pollution rates.

**Fig 3:**
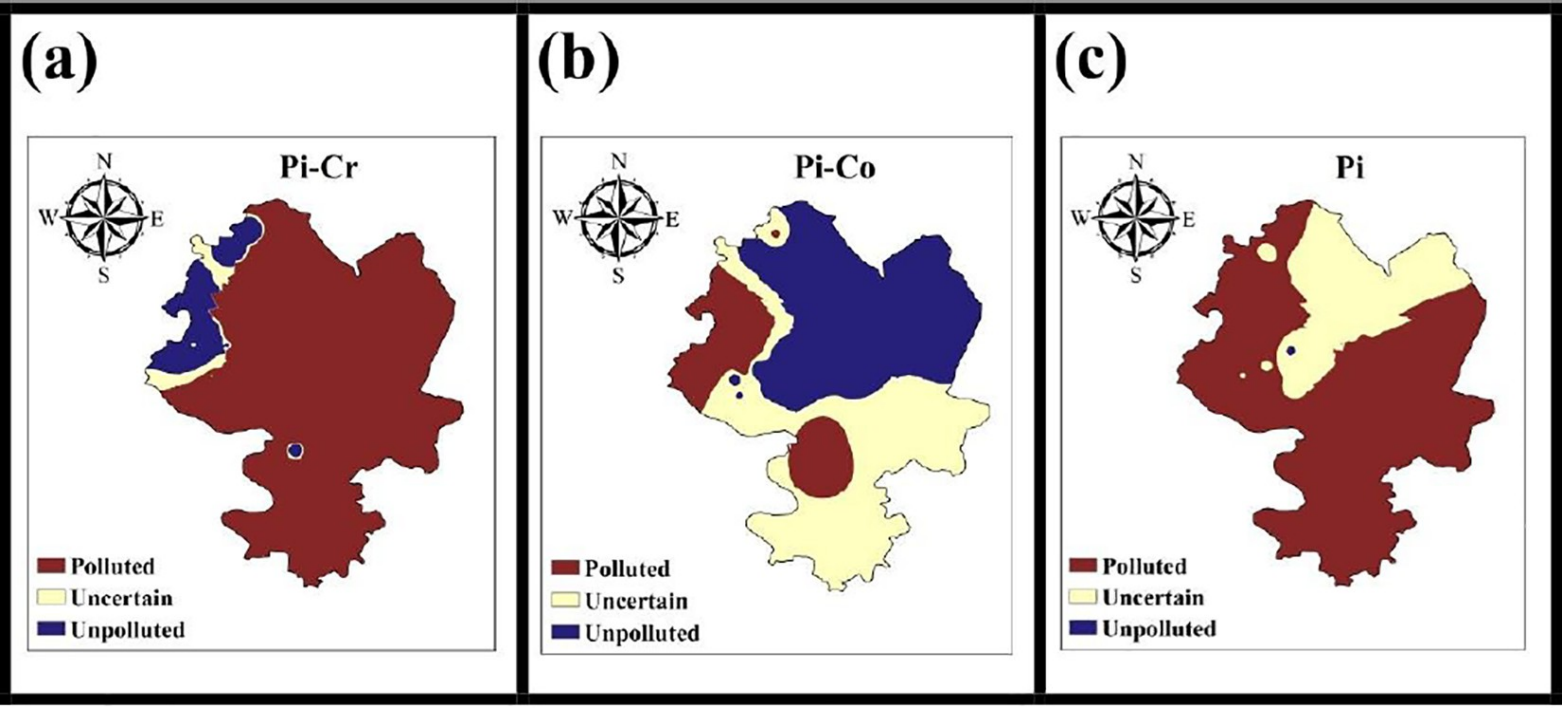
Detection of pollution areas for (a) Pi-Cr, (b) Pi-Co and (c) integrated Pi.

The analysis on a per metal basis using Pi to identify areas of pollution, as shown in Fig 3, shows significant results. The use of the combined kriging and IDW interpolation method to identify polluted areas resulted in a remarkable increase in the identification of soil pollution by Cr to 90.53%, exceeding the statistical result by 37.2% (Table 3 and Fig 3A). Similarly, soil contamination for As increased by 13.25%, from 83.33% to 96.58%. For Co, on the other hand, the combined interpolation analysis showed a lower percentage of contaminated areas, namely a decrease of 30.1% compared to the statistical result (Table 3 and Fig 3B). In addition, minor differences were found between the statistical and combined interpolation methods in the identification of contaminated areas, with negligible uncertainty for Mn, Cu and Zn. This can be attributed to the widespread enrichment of these metals at most sites. Cu and Zn were generally considered to be of no concern, with only a fraction of sites identified as contaminated. Using the combined method, 0.24% and 0.22% contaminated areas were identified.

**Table 3:** Detection of pollution areas with statistical location calculation and combined interpolation model.

Metal	Statistical site calculation (%)	Combined interpolation (%)
Pb	100	100
Cr	53.33	90.53
Mn	96.67	99.94
Cu	3.333	0.24
Zn	10	0.22
Co	43.33	13.23
Ni	0	0
As	83.33	96.58
All metal(loid)s	66.67	79.17

Fig 3C illustrates the results of the identification of pollution areas for all heavy metals (loids) using integrated Pi. It shows that 79.17% of the area was identified as contaminated, an increase of 12.5% compared to the assessments based solely on on-site calculations. The spatial distribution indicates that the contaminated areas were predominantly located on commercial and agricultural land. In contrast, the uncontaminated areas, which accounted for 3.2% of the study area, were located in the floodplains and educational areas. A notable observation is the significantly lower proportion of clean areas in this combined result compared to the combined results of the interpolation and the site-based calculation for Pi. This discrepancy is primarily due to the higher classification of unsafe areas, which accounted for 17.63% in the combined method.

In the study area, the soils contaminated with Pb, Cr, Mn and As were the most polluted. In contrast, the soils in many regions showed a medium to high pollution capacity (Pi>0.7) for Cu, Zn, Co and Ni. Although the combined method for identifying contaminated areas showed only small deviations from the statistical results for Mn, Cu and Zn, the discrepancies for Cr and Co were more pronounced. These discrepancies may be due to the occurrence of the specific metals in contaminated or uncontaminated areas. The significant proportion of uncertain areas, particularly in the case of Co, highlights the need to include additional sampling sites to improve interpolation accuracy. Furthermore, these unsafe areas need to be prioritized to prevent their transition to polluted regions.

### 3.4 SOM analysis for spatial pattern detection

**Fig 4:**
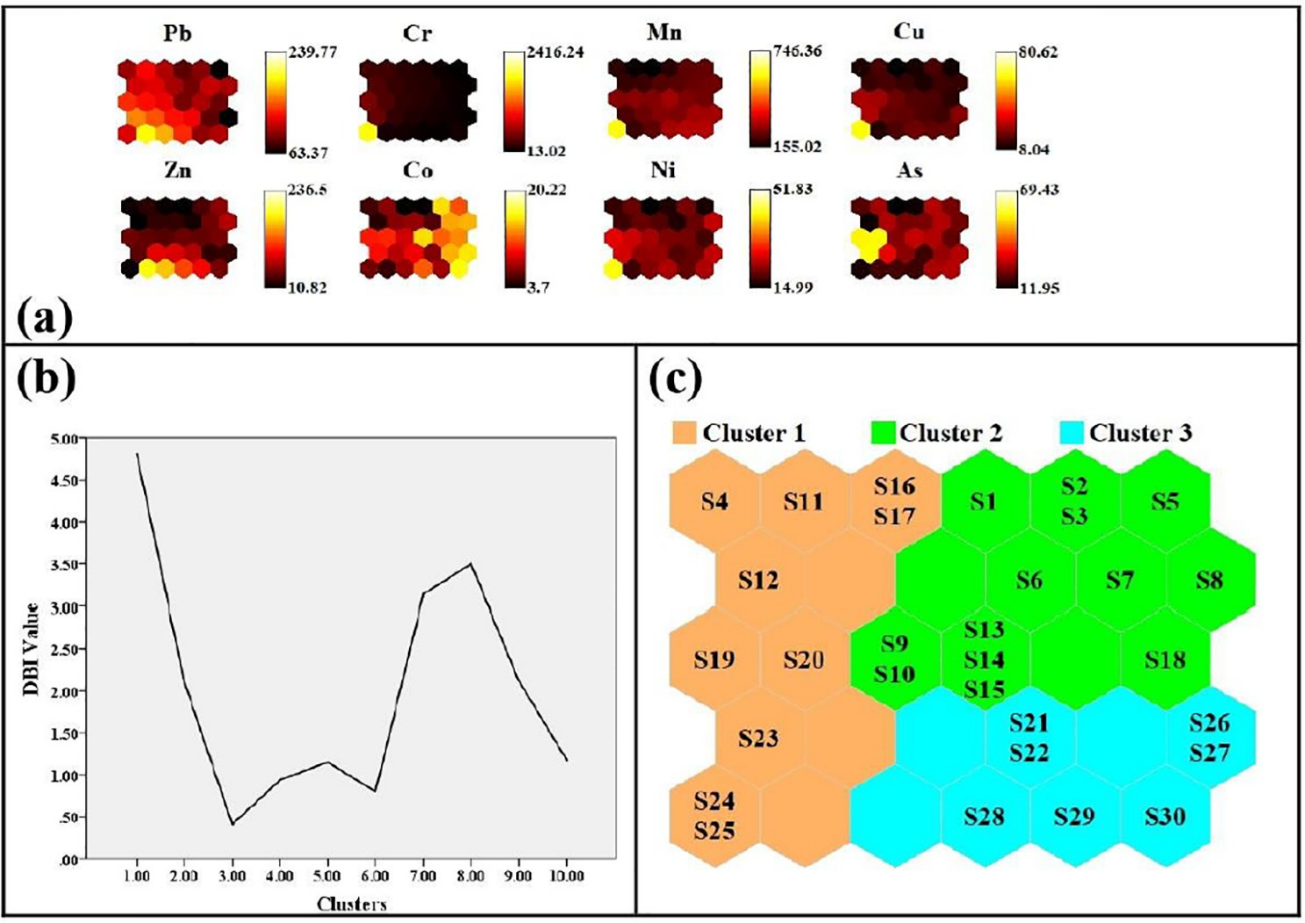
Analysis of the self-organizing map, (a) SOM component levels, (b) DBI values for different clusters SOM clustering for sampling points.

The visual representation of the results of the SOM analysis can be seen in Fig 4, in which the sample locations are divided into three different clusters by color. Fig 4A shows the SOM maps for the eight parameters of heavy metals and metalloids resulting from the analysis. Each SOM matrix map represents an index value determined during the pre-processing phase of the data [32] and is represented by different shades of yellow and black. These SOM matrix maps visually represent both informational and esthetic relationships between parameters through specific color variations. Smaller hexagonal areas on the SOM plane indicate greater similarity between the measured features. In addition, similar and dissimilar colors on the SOM plane correspond to positive and negative relationships between the metalloids, respectively.

Three-color spatial patterns were identified that resemble eight metal(loid)s for different land-use soils. The first spatial pattern shows neurons arranged in ascending order from bottom to top left. Pb and Zn have comparable color patterns and show a significant positive correlation. This spatial pattern is due to the effects of unprocessed waste from the study area, burning brickfields and untreated industrial waste released into the soil environment adjacent to the study area [49,54]. Cr, Cu and Ni fit the second spatial pattern where the neurons were arranged in decreasing order from bottom to top and showed an identical pattern, suggesting that extensive anthropogenic fertilizers and pesticides used in agrochemical plants are responsible for controlling the environmental quality of the soil in the study area [55]. The third spatial pattern shows that the maximum and minimum percentage of neurons are arranged from the right corner to the left corner with Co, As and Mn having their negative association, suggesting a common lithogenic source [20]. Interestingly, the contamination sources of heavy metal(loid)s in the different land-use soils derived from the SOM analysis showed a consistent distribution pattern of metal(loid)s in the PMF models.

The determination of the appropriate number of clusters for SOMs, ranging from 1 to 10 clusters, was based on the calculation of Davies-Bouldin Index (DBI) values after the training phase. The lowest DBI value obtained (0.43 in cluster 3) represents the optimal cluster membership (Fig 4B). As shown in Fig 4C, the different soil samples with different land use were then divided into three clusters: Cluster 1 comprised ten samples (S4, S11-12, S16-17, S19-20, S23-25), Cluster 2 thirteen samples (S1-3, S5-10, S13-15 and S18) and Cluster 3 seven samples (S21-22, S26-30). In each cluster, there was at least one contaminant with significantly higher metal contents than in the others. In particular, elevated concentrations of Cr and Ni were mainly observed in cluster 1, which is consistent with the observed correlation value between these two metals. It is noteworthy that the sites characterized by high Cu concentrations in the soil coincide with those showing elevated Ni concentrations in the SOM, especially the sample sites (S19, S24 and S25) in cluster 1, suggesting an agrochemical source. Conversely, the distribution pattern of Co in the SOM showed remarkable differences. Higher Co and Mn contents were mainly found in cluster 2, suggesting natural sources. Cluster 3 belongs to the elevated levels of Pb and Zn, indicating industrial discharges. From the SOM analysis it can be concluded that the clusters represent a maximum of sources for identification and allocation.

### 3.5 Quantifying source of soil heavy metal(loid)s

Source identification using the PMF model has recently attracted considerable attention in the scientific community as a reliable receptor method for determining contaminant concentrations and sources in the soil environment [14,20]. In the EPA PMF 5.0 model, the input files consisted of concentration data for eight metal(loid)s in thirty soil samples and the corresponding uncertainty data. To optimize the results, the number of factors was varied between 3, 4, and 5, while the “random start seed number” option was selected, and 20 runs were performed. After 20 iterations with the aim of minimizing the Q value, the optimal number of factors was identified as three, as shown in S3 Fig in S1 File. S6 Table in S1 File shows the coefficient of determination (R2) between the determined and projected values, ranging from 0.204 (Mn) to 0.988 (Zn), indicating a significant correlation for most metals except Mn. Consequently, the results of the PMF method for the assessed heavy metals were considered reliable and widely used. It should be noted that in PMF analysis, minimizing the Q value usually gives the most accurate results, residuals are usually in the range of 3 to -3, and higher correlation coefficients (R2) are obtained when comparing actual and predicted values [34,56].

**Fig 5:**
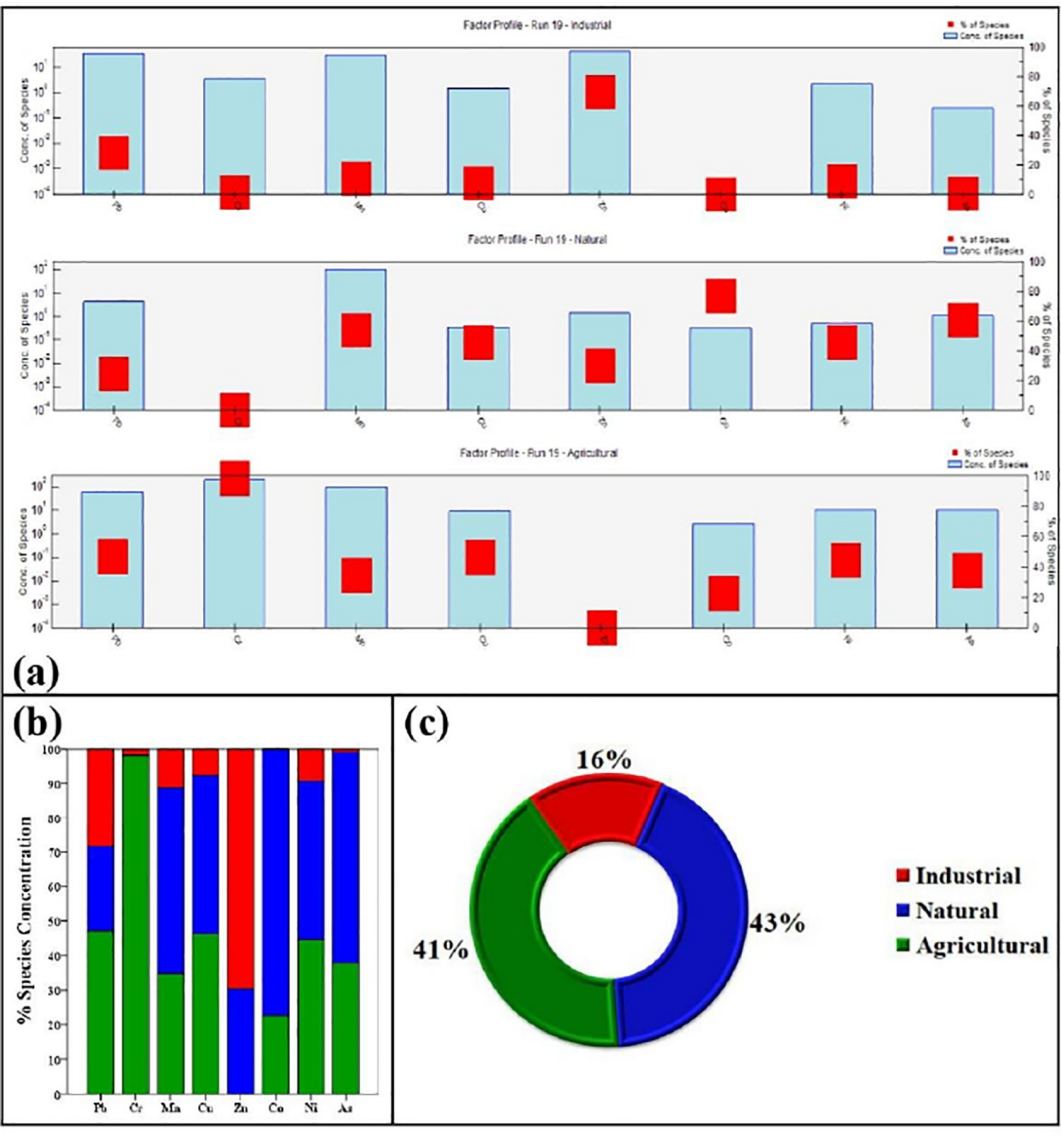
Profiles and contributions of sources of heavy metal(loid)s (a). Explained variation of factors calculated with the PMF model; (b) and total factor fingerprints of heavy metal(loid)s in the soil of the study area (c).

The results of the PMF model (Fig 5A and 5B and S7 Table in S1 File) show that Zn (69.62%) and Pb (28.34%) are the dominant factor 1 (industry), contributing the least (16%) to the metal concentrations in the soil of the study area. Factor 1, on the other hand, contributed little to Cr (1.81%), Ni (9.25%) and As (0.96%). The soils from industrial effluents in Chilmari also showed that the main source of Zn and Pb input was anthropogenic industrial activities. In recent decades, small workshops that produced and processed metal, carried out dyeing and lining processes, galvanized and smelted metal have gained importance in the area. In the absence of strict pollution legislation and enforcement, regular emissions of metal contaminants into the environment have led to their accumulation in soils and sediments. As a result, Factor 1 is thought to originate mainly from industrial point sources.

Factor 2 (natural), which was mainly dominated by Co (77.43%), As (61.15%) and Mn (54.11%) and contributed less to Cr, Pb and Zn (<40%), was responsible for the highest 43% of total metal concentrations (Fig 5C). Factor 2 revealed that Cr and Zn contributed only about 30.46%. Various applications, such as abnormal activities towards nature, are the reason for this partitioning of metals. In addition, studies have shown that Co and Ni in soil often have a geologic origin as they are derived from natural weathering of the parent material of the soil [33,55]. Most sites showed concentrations comparable to the natural background levels of the soil. Excessive inputs of agrochemicals to support agricultural activities were also found in agricultural soils. Previous studies on the PMF method have also found high levels of As pollution from agricultural sources due to comparatively low efficiency in the use of fertilizers and pesticides, leading to accumulation in the environment. The conclusion from the study shows that agricultural sources are the second largest source of TM in the soils of the study area.

Factor 3 (agriculture) was responsible for 41% of the metal concentrations in the soil; the highest loading (70–90%) was found for Cr, Ni, Pb and Cu on factor 3. This source has a minimal contribution from Zn and Co (<30%). Cr is often present in agricultural inputs and enters the soil during land application, where it can accumulate to critical levels during successive applications [11]. Pesticides can contain heavy metals such as As, Pb and Cu as active components or impurities. Heavy metals such as As, Cr and Pb also enter the soil via human waste and industrial effluents from wastewater irrigation [57]. In addition, organic fertilizers derived from animal waste or composted organic matter may contain heavy metals such as Pb originating from animal feeding or composting. Therefore, factor 3 can be associated with agricultural activities.

**Fig 6:**
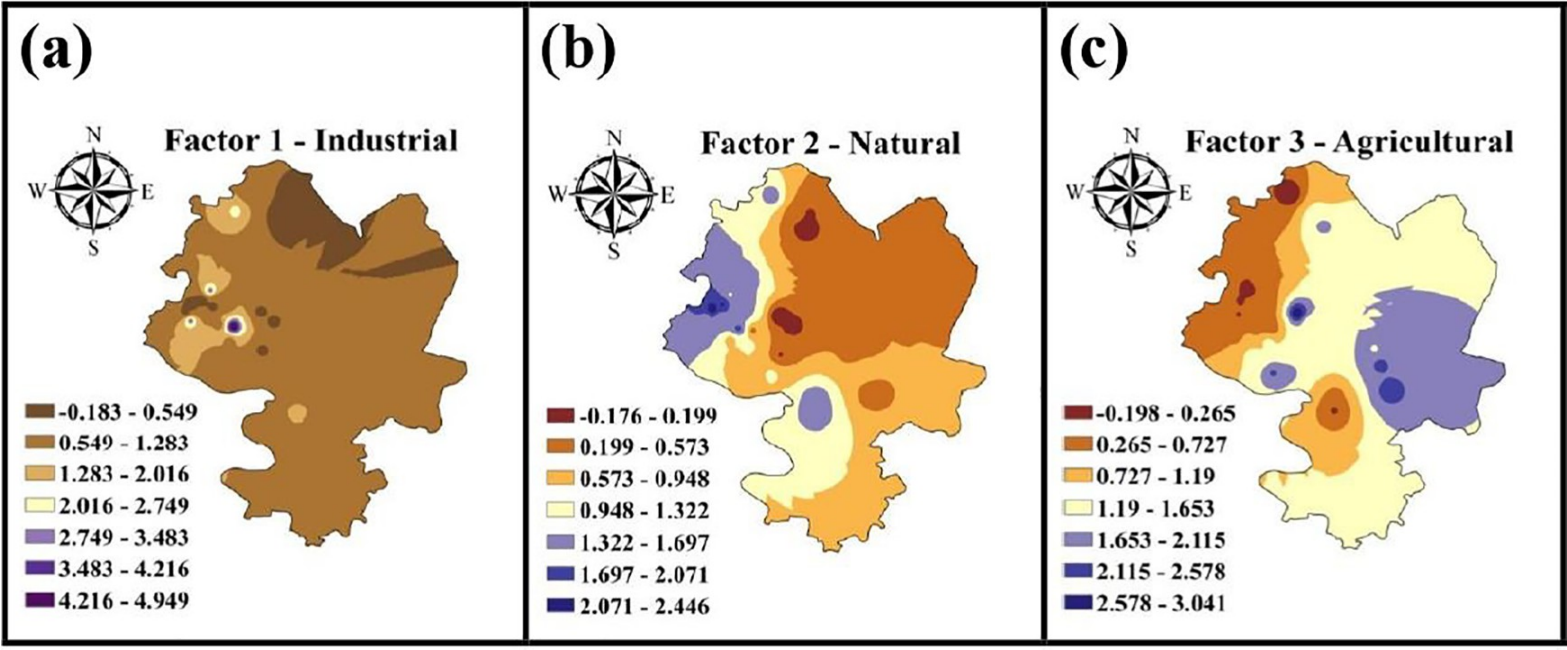
Intensities for source factors of heavy metal(loid)s contents in soil, determined by the PMF model.

Using the IDW interpolation method, the spatial intensity of the source factors from the PMF method was visualized based on their contributions to the metal concentrations (Fig 6). Regional potential industrial emissions (Fig 6A) showed higher contributions to metal (loid) concentrations in soil in the northwestern part of the study area, while lower contributions were observed in the southwest. This pattern can be attributed to small industrial complexes with unregulated metal emissions in these areas. Studies by Habib et al. (2023) and Proshad et al. (2023) have shown a significant contribution of industrial activities to metal risks in the adjacent ecological communities, especially in the building land, forest and farmland areas identified by the PMF method [14,34]. In contrast to factors 2 and 3, factor 1 showed high spatial variability (-0.183 to 4.949), indicating a less identical spatial distribution pattern at the regional scale, in contrast to the distribution of the parent material in the soil. Factor 2 was particularly strongly associated with Co and showed a clear “hot spot” in the north (Fig 6B). In addition, the overlaid intensities for factor 2 showed some similarity with the identified pollution areas (Fig 3B). Conversely, agricultural sources predominated in the south-eastern part of the study area and showed less spatial variability, probably influenced by soil and river characteristics (Fig 3C).

## 4. Implication for environmental management of soil heavy metal(loid)s

Contamination with heavy metals lead to a deterioration in the environmental quality of the soil. It is a major problem due to its high ecotoxicity, long-term persistence and bioaccumulation through the food web, with adverse effects on ecology and human health. In this article, the EPC was used to assess the heavy metal load in different soils. The critical load range was determined based on a coupled spatial analysis and critical uncertainty assessment. The results show that bedrock plays a central role in the accumulation of Ni, Cr and As in different soils. Based on the pollution risk analysis, the PMF and SOM models were used in conjunction with a spatial assessment to quantitatively evaluate potential pollution sources of heavy metal(loid)s in the study region.

This study recommends that the port area of Chilmari be regularly monitored for heavy metals in the various soils. An ecological remediation strategy by the government of Bangladesh and non-governmental organizations is needed to assess the role of the Ministry of Environment in causing the rapid increase in heavy metal pollution in the Chilmari port area and to enact new stringent laws to stop the uncontrolled discharge of industrial pollutants from point sources into the suburban soil ecosystem. These findings are helpful for the prevention and reduction of heavy metal pollution in soils through various efficient measures in Chilmari port area. Our research also suggests that the geochemical speciation of toxic metals in the soil environment of a suburban study area in the northern region of Bangladesh should be systematically and consistently studied and assessed to ensure an environmental sustainability perspective. Besides the basic information provided by our study, the environmental authorities could keep an eye on the soil ecosystems to protect the health of the residents and assess ecological insurance claims from the past.

## 5. Conclusions

This study deals with the investigation and interpretation of pollution risks and critical regions of heavy metals and metalloids in soil in different land use types. It aims to shed light on the sources of metal pollution and identify spatial patterns. The results show that the average concentrations of Zn, Cu, Mn, Co and Ni in the soils of the study area are below background levels. In contrast, the average concentrations of Cr, Pb and As exceed background levels, indicating a significant risk to ecology and human health. The soil EPC primarily reflects heavy metal contamination by Pb and Cr and underlines the urgency of priority control measures and early risk detection for these metals. The comparison between site-specific calculations and spatial analysis of metal-contaminated areas shows that uncertainties vary. The application of combined methods to determine critical pollution regions shows significant contamination by Pb and Cr with minimal uncertainty ranges (0.01% and 0.3% respectively).

In contrast, very uncertain regions (17.63%) were identified for co-contamination. The uncertainty was mainly related to the spatial pattern of the predominant metals. The ANOVA test revealed that Cu, Cr and Ni levels were significantly higher in commercial, floodplain and agricultural soils than in residential and school soils. In addition, the Pb and Zn content in the soil of residential, commercial and school soils was higher than that of agricultural and floodplain soils, while land use patterns had no effect on the concentrations of As, Ni and Cr. The results of the SOM assessment in this article agreed well with the results of the PMF model. The PMF model identified three distinct metal(loid) sources within the study area, which together accounted for 43% (factor 1), 41% (factor 2) and 16% (factor 3) of the total variance. The analysis of heavy metal pollution characteristics and spatial distribution revealed that the intensity of sources originated from industrial point emissions, agrochemical application and natural geogenic processes. Considering the ecological hazards and the persistent presence of heavy metals in soil ecosystems, the implementation of strict environmental regulations and measures is crucial to curb the accumulation of metal(loids) in different soil types. This requires continuous monitoring and control of anthropogenic metal(loid) emissions and the implementation of effective soil remediation strategies to ensure ecological integrity and food safety.

## Supporting information

S1 Data (XLSX)

S1 File S1–S3 Figs, S1–S7 Tables. (DOCX)
