# Supplementary material for: Innovative strategies for pollution assessment in Northern Bangladesh: Mapping pollution areas and tracing metal(loid)s sources in various soil types
Source: PLoS One. 2025 Feb 3;20(2):e0311270. doi: 10.1371/journal.pone.0311270 (PMC11790134; doi:10.1371/journal.pone.0311270)
Supplement: S1 File — (DOCX) [file pone.0311270.s002.docx]

**Supplementary Materials**

**Table S1** Classification standards for the comprehensive assessment of heavy metal exposure indices

| Grade | A | B | C | D | E |
| --- | --- | --- | --- | --- | --- |
| Ecological Risk index | <30 | 30≤ ER<50 | 50≤ ER<100 | 100≤ ER<150 | ≥150 |
| Nemerow pollution index | <0.7 | 0.7≤ NPI<1 | 1≤ NPI<2 | 2≤ NPI<3 | ≥3 |
| Environmental carrying capacity | ˃1 | 1≥Pi˃0.7 | 0.7≥Pi˃0.3 | 0.3≥Pi˃0 | ≤0 |
| Pollution description | Safe | Precaution | Slight polluted | Moderate polluted | Heavy polluted |

**Table S2.** Global geochemical background values and toxic response factor values of the heavy metal(loid)s

|  | **Reference** | **Pb** | **Cr** | **Mn** | **Cu** | **Zn** | **Co** | **Ni** | **As** |
| --- | --- | --- | --- | --- | --- | --- | --- | --- | --- |
| Risk screening value (Cs) | 3 | 90 | 150 | 130 | 50 | 200 | 20 | 70 | 25 |
| Background value (Cb) | 1 | 20.00 | 90.00 | 850 | 45.00 | 95.00 | 10.00 | 68.00 | 15.00 |
| Toxic response (Tr) | 2 | 5.00 | 2.00 | 1.00 | 5.00 | 1.00 | 5.00 | 5.00 | 10.00 |
| Standard limit (Sj) (mg/kg) | 4* | 50 | 100 | 2000 | 100 | 300 | 50 | 100 | 20 |

*WHO and FAO standard

**Table S3**. Method detection limit (MDL) for heavy metal analysis

| **Element Name** | **Method detection limit (mg/kg)** |
| --- | --- |
| Pb | 0.25 |
| Co | 0.12 |
| Zn | 0.21 |
| Cu | 0.05 |
| Ni | 0.54 |
| As | 0.0003 |
| Mn | 0.15 |
| Cr | 0.16 |

**Table S4** Statistics for integrated Nemerow pollution index (NPI), ecological risk (ER) and environmental carrying capacity (Pi)

| Index | Max | Min | Average | SD | Median |
| --- | --- | --- | --- | --- | --- |
| ER | 116.99 | 41.51 | 70.50 | 21.84 | 65.36 |
| NPI | 3.70 | 0.44 | 0.98 | 0.61 | 0.77 |
| Pi | 4.90 | -3.82 | 3.44 | 1.64 | 3.97 |

**Table S5** Correlation analysis between the heavy metal(loid)s of different land uses in the soils of Chilmari

|  |  | **Pb** | **Cr** | **Mn** | **Cu** | **Zn** | **Co** | **Ni** | **As** |  |
| --- | --- | --- | --- | --- | --- | --- | --- | --- | --- | --- |
| **Residential Area** | **Pb** | 1 | .417 | -.056 | .175 | .835* | -.586 | .336 | -.261 | **Commercial Area** |
|  | **Cr** | -.030 | 1 | .211 | .157 | .109 | .235 | .206 | .547 |  |
|  | **Mn** | .478 | .392 | 1 | .736 | .254 | .072 | .712 | .373 |  |
|  | **Cu** | .471 | .446 | .933* | 1 | .444 | .237 | .985** | .490 |  |
|  | **Zn** | .777 | -.534 | .447 | .360 | 1 | -.684 | .569 | -.315 |  |
|  | **Co** | .005 | .572 | -.401 | -.304 | -.584 | 1 | .105 | .847* |  |
|  | **Ni** | .323 | .334 | .030 | .364 | -.082 | .421 | 1 | .397 |  |
|  | **As** | -.569 | .644 | .309 | .413 | -.624 | -.008 | .155 | 1 |  |
| **Educational Area** | **Pb** | 1 | .566 | .448 | .522 | -.135 | .290 | .529 | -.331 | **Floodplain Area** |
|  | **Cr** | -.043 | 1 | .983** | .997** | -.105 | .687 | .996** | -.238 |  |
|  | **Mn** | -.812* | .214 | 1 | .993** | -.014 | .763* | .990** | -.112 |  |
|  | **Cu** | -.666 | .691 | .845* | 1 | -.091 | .711 | .999** | -.201 |  |
|  | **Zn** | .968** | -.270 | -.775 | -.768 | 1 | .485 | -.083 | .873* |  |
|  | **Co** | -.741 | -.312 | .637 | .308 | -.619 | 1 | .719 | .502 |  |
|  | **Ni** | -.635 | .602 | .830* | .933** | -.714 | .509 | 1 | -.183 |  |
|  | **As** | -.489 | .350 | .546 | .638 | -.552 | .462 | .780 | 1 |  |
| **Agricultural Area** | **Pb** | 1 |  |  |  |  |  |  |  |  |
|  | **Cr** | .587 | 1 |  |  |  |  |  |  |  |
|  | **Mn** | -.069 | .489 | 1 |  |  |  |  |  |  |
|  | **Cu** | .516 | .680 | .761 | 1 |  |  |  |  |  |
|  | **Zn** | .300 | .740 | .898* | .946* | 1 |  |  |  |  |
|  | **Co** | .271 | .574 | .917* | .950* | .965** | 1 |  |  |  |
|  | **Ni** | .400 | .752 | .854 | .975** | .993** | .965** | 1 |  |  |
|  | **As** | .538 | .716 | .796 | .922* | .902* | .923* | .927* | 1 |  |

*. Correlation is significant at the 0.05 level (2-tailed). **. Correlation is significant at the 0.01 level (2-tailed).

**Table S6** Validation of PMF Model (Regression diagnostics)

|  |  |  |  |  | KS Test | |
| --- | --- | --- | --- | --- | --- | --- |
| Species | Intercept | Slope | SE | R^2^ | Stat | P Value |
| Pb | 1.812 | 0.968 | 18.280 | 0.811 | 0.118 | 0.796 |
| Cr | 131.427 | 0.271 | 107.426 | 0.555 | 0.272 | 0.024 |
| Mn | 188.968 | 0.290 | 61.341 | 0.204 | 0.129 | 0.699 |
| Cu | 14.043 | 0.260 | 3.241 | 0.542 | 0.114 | 0.828 |
| Zn | 2.506 | 0.952 | 5.366 | 0.988 | 0.143 | 0.575 |
| Co | 0.788 | 0.913 | 1.399 | 0.920 | 0.124 | 0.750 |
| Ni | 8.929 | 0.617 | 3.313 | 0.660 | 0.260 | 0.035 |
| As | 16.172 | 0.338 | 5.340 | 0.548 | 0.122 | 0.764 |

**Table S7** Source contributions for metals in different land use soils by the PMF model

|  | Industrial | Natural | Agricultural |
| --- | --- | --- | --- |
| Pb | 28.34 | 24.56 | 47.10 |
| Cr | 1.81 | 0.08 | 98.11 |
| Mn | 11.13 | 54.11 | 34.76 |
| Cu | 7.70 | 45.93 | 46.37 |
| Zn | 69.62 | 30.38 | 0.00 |
| Co | 0.00 | 77.43 | 22.57 |
| Ni | 9.25 | 46.04 | 44.71 |
| As | 0.96 | 61.15 | 37.89 |


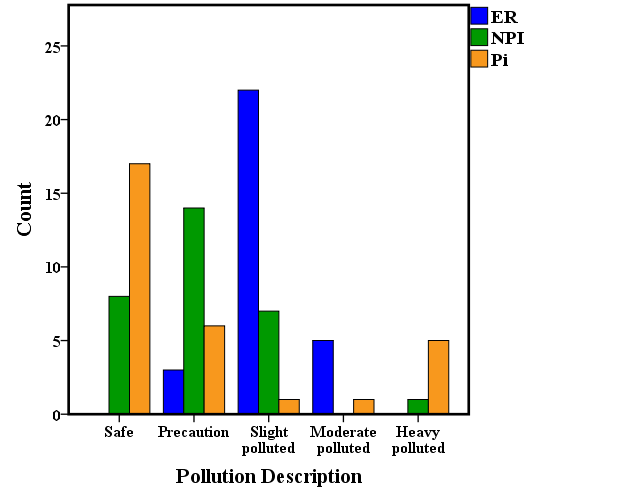


**Fig. S1** Distribution of site pollution risk for integrate Nemerow pollution index (NPI), ecological risk (ER) and environmental carrying capacity (Pi).


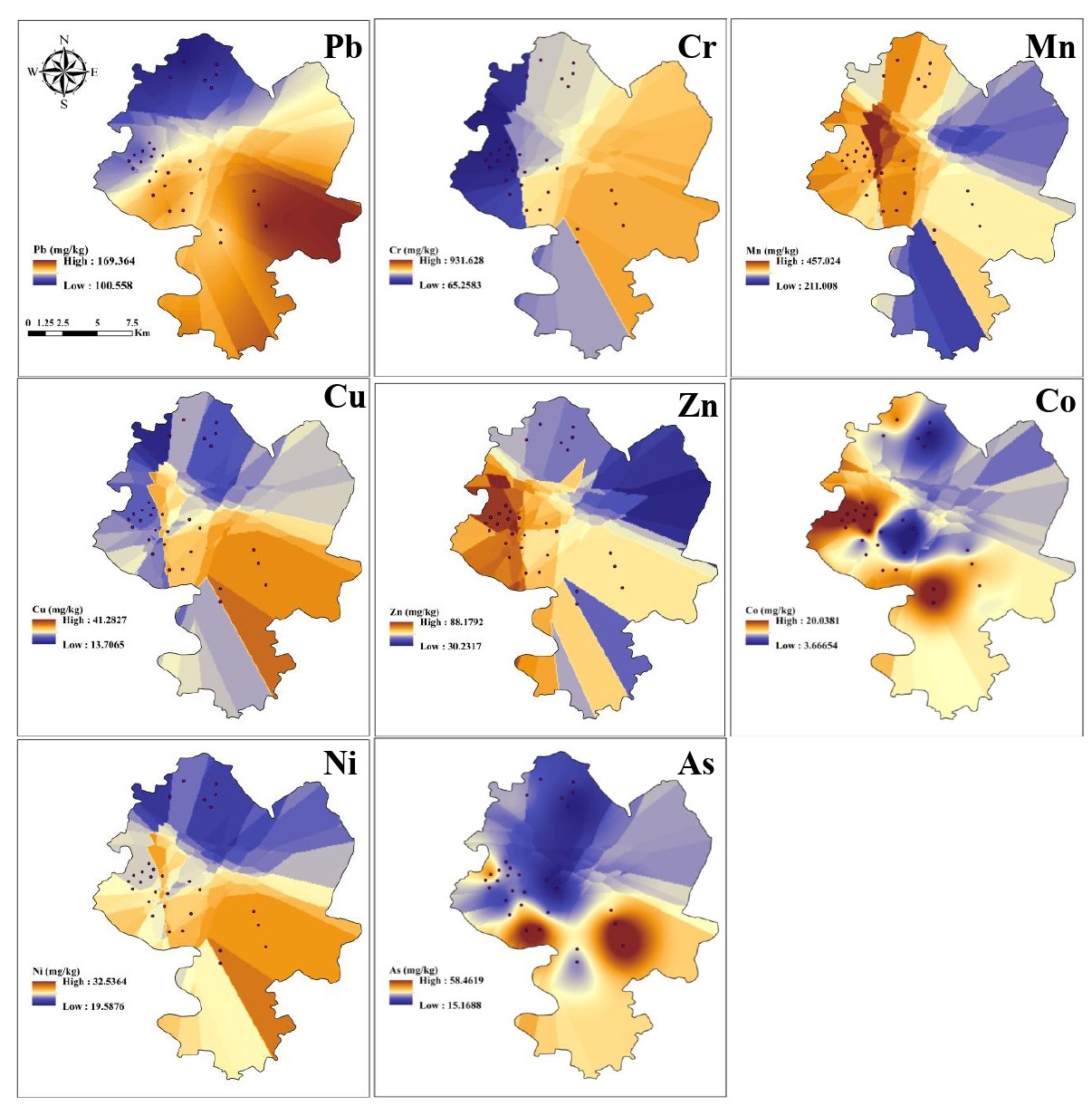


**Fig. S2** Spatial distributions of heavy metal(loid)s in soil based on an empirical Bayesian kriging model


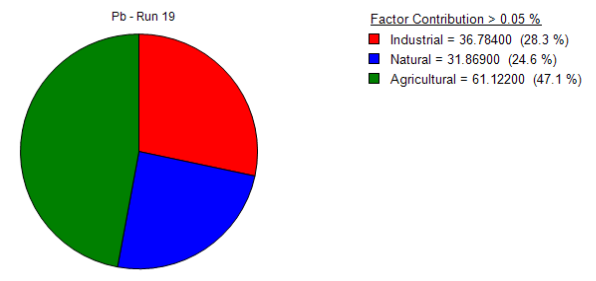

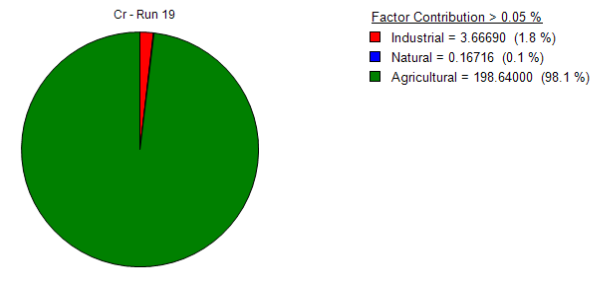

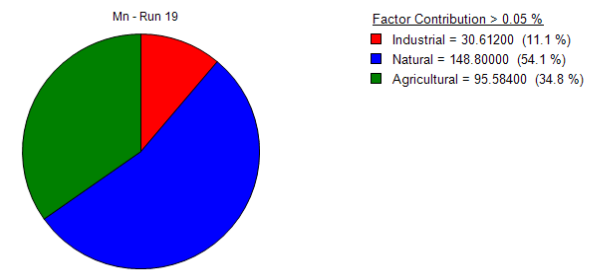

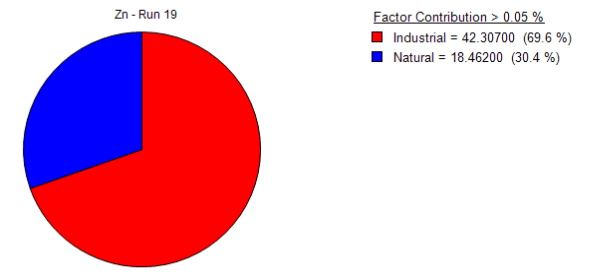

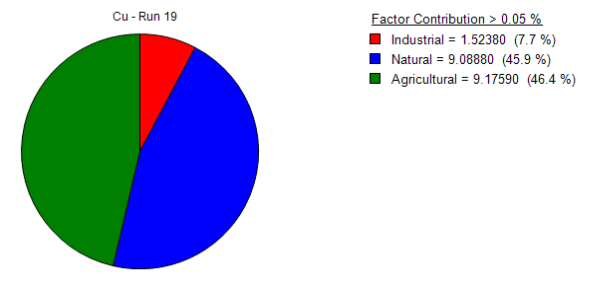

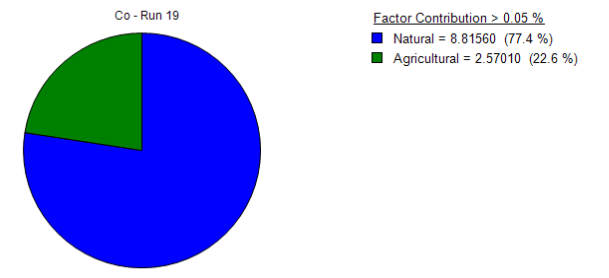

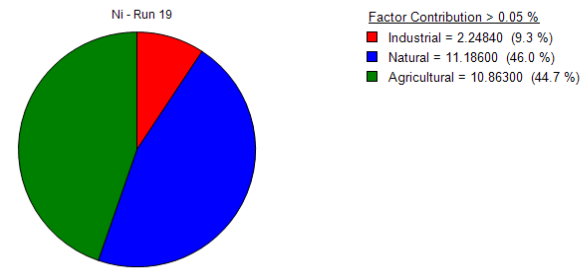

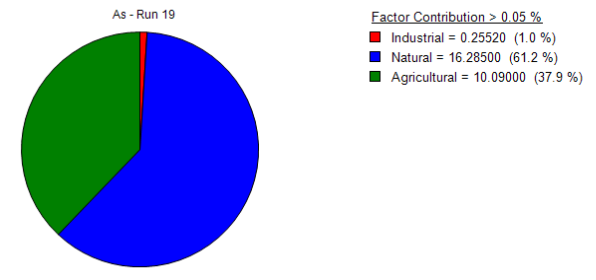


**Fig. S3** Source contribution for individual metals

References

Turekian, K. K., & Wedepohl, K. H. (1961). Distribution of the elements in some major units of the earth's crust. *Geological society of America bulletin, 72*(2), 175-192.

Edori, O. S., & Kpee, F. (2017). Index models assessment of heavy metal pollution in soils within selected abattoirs in Port Harcourt, Rivers State, Nigeria. *Singapore Journal of Scientific Research, 7*(1), 9-15.

Xu, C., Pu, J., Wen, B., & Xia, M. (2021). Potential ecological risks of heavy metals in agricultural soil alongside highways and their relationship with landscape. *Agriculture*, *11*(8), 800.

Chiroma, T. M., Ebewele, R. O., & Hymore, F. K. (2014). Comparative assessment of heavy metal levels in soil, vegetables and urban grey waste water used for irrigation in Yola and Kano. *International refereed journal of engineering and science*, *3*(2), 01-09.
